# Antifungal susceptibility profiles of *Candida* and non-*albicans s*pecies isolated from pregnant women: implications for emerging antimicrobial resistance in maternal health

**DOI:** 10.1128/spectrum.00787-25

**Published:** 2025-06-02

**Authors:** George Aboagye, Sayanika Waikhom, Emmanuel Akomanin Asiamah, Clement Okraku Tettey, Hintermann Mbroh, Cecilia Smith, George Yiadom Osei, Karikari Asafo Adjei, Richard Harry Asmah

**Affiliations:** 1 Department of Nutrition and Dietetics, School of Allied Health Sciences, University of Health and Allied Sciences, Ho, Volta Region, Ghana; 2 Department of Biomedical Sciences, School of Basic and Biomedical Sciences, University of Health and Allied Sciences, Ho, Volta Region, Ghana; 3 Department of Medical Laboratory Sciences, School of Allied Health Sciences, University of Health and Allied Sciences, Ho, Volta Region, Ghana; 4 Department of Obstetrics and Gynaecology, Ho Teaching Hospital 607452 , Ho, Volta Region, Ghana; 5 Department of Microbiology, Ho Teaching Hospital 607452 , Ho, Volta Region, Ghana; Universidade de Sao Paulo, Sao Paulo, Brazil

**Keywords:** antifungal susceptibility, *Candida *species, vulvovaginal candidiasis, pregnancy, antifungal resistance, maternal health, antenatal care

## Abstract

Vulvovaginal candidiasis (VVC) is a common fungal infection, affecting approximately 75% of women at least once in their lifetime. *Candida albicans* is the predominant pathogen, responsible for 85%–90% of symptomatic cases. This study assessed the prevalence and antifungal susceptibility patterns of *Candida* species isolated from pregnant women with VVC to determine species-specific resistance trends and treatment implications. A cross-sectional study was conducted between November 2020 and July 2023 at Ho Teaching Hospital and Ho Municipal Hospital, the primary referral health facilities in the Volta Region. A total of 205 pregnant women across all trimesters participated. Vaginal swabs were collected and cultured for *Candida* species isolation and identification. Antifungal susceptibility of the isolates was assessed using the Kirby-Bauer disk diffusion method against clotrimazole (10 µg), fluconazole (10 µg), itraconazole (10 µg), and nystatin (100 IU). The prevalence of VVC among participants was 50.7%. *C. albicans* was most frequent (22.9%), followed by *Candida krusei* (14.1%), *Nakaseomyces glabrata* (9.3%), *Candida tropicalis* (2.4%), and *Candida dubliniensis* (2.0%). *C. albicans* exhibited high susceptibility to clotrimazole (87.2%) and nystatin (87.2%) but lower susceptibility to fluconazole (44.7%) and itraconazole (44.7%). Resistance was observed, particularly in *C. dubliniensis* to fluconazole (100%). The findings highlight the diversity of *Candida* species in VVC and the necessity of species-specific antifungal therapy. Laboratory diagnostics are critical to guide treatment and mitigate antifungal resistance. Routine surveillance and judicious antifungal use are vital to preventing resistance, while antenatal visits offer opportunities for education on VVC prevention and management.

**IMPORTANCE:**

Vulvovaginal candidiasis (VVC) is a common yeast infection that affects many women, especially during pregnancy. This study examined how often VVC occurs in pregnant women and how well different antifungal treatments work. The researchers who studied 205 pregnant women in Ho, Volta Region, Ghana, found that over half (50.7%) had VVC. The most common fungus was *Candida albicans*, but other types, such as *Nakaseomyces glabrata* and *Candida krusei*, were also present—and some were resistant to common antifungal drugs. While clotrimazole and nystatin were effective, many fungi were resistant to fluconazole and itraconazole medications, which are often used to treat infections. Since untreated VVC can lead to complications such as preterm birth, the study emphasizes the need for early testing and tailored treatments to prevent health risks, the importance of routine screenings during pregnancy, and better awareness about safe and effective antifungal treatments.

## INTRODUCTION

Vulvovaginal candidiasis (VVC), also referred to as *Candida* vaginitis, is a fungal infection caused by *Candida* species in the vaginal region. It is characterized as an infection confined to the estrogenic vagina and vestibulum, with the potential to spread to the intercrural region and the external surfaces of the labia minora and majora (1). VVC is the second most common cause of vaginal infection after bacterial vaginosis, with approximately 90% of cases attributed to *Candida albicans* (2).

In the United States, VVC ranks as the second most frequent vaginal infection following bacterial vaginosis, whereas in Europe, it remains one of the leading causes of vaginitis (3). It is estimated that 75% of women experience at least one episode of VVC in their lifetime, with 40%–50% encountering a second episode and approximately 7%–8% developing recurrent VVC (4). The condition is characterized by symptoms such as pruritus, vaginal discomfort, dysuria, dyspareunia, and abnormal vaginal discharge, which significantly affect the quality of life of affected women (5).

Pregnant women are particularly susceptible to VVC due to several physiological changes that create a favorable environment for *Candida* overgrowth. These changes include increased estrogen levels, enhanced vaginal glycogen synthesis, and immunologic alterations, all of which promote the proliferation and persistence of *Candida* species in the vaginal mucosa (6, 7). Studies estimate that 10%–15% of asymptomatic women are colonized with *Candida*, while 70%–75% of women will experience at least one episode of VVC in their lifetime (8). However, among pregnant women, prevalence rates are notably higher, ranging from approximately 20% to 40% (9–11), with even higher rates exceeding 60% reported in certain populations (12). This reflects the increased susceptibility associated with pregnancy-related physiological changes.

Although *C. albicans* remains the predominant causative species, recent studies have reported an increasing prevalence of non-*albicans Candida* (NAC) species, including *Nakaseomyces glabrata*, *Candida tropicalis*, *Candida krusei*, and *Candida dubliniensis* (13–15). This shift toward NAC species is concerning, as these species often exhibit reduced susceptibility to azole antifungal agents, which are the first-line treatment for VVC (16). Studies indicate that many women who self-diagnose and self-medicate for presumed VVC may not actually have the infection, thereby increasing the risk of antifungal resistance among *Candida* species (17, 18).

Beyond its impact on maternal health, untreated VVC during pregnancy has been associated with several adverse pregnancy outcomes, including preterm birth, premature rupture of membranes, chorioamnionitis, and neonatal *Candida* colonization, which may lead to systemic infections in newborns (19–21). Vertical transmission of *Candida* can occur during vaginal delivery, resulting in neonatal oral thrush, diaper dermatitis, and, in severe cases, invasive candidiasis (22). Additionally, horizontal transmission may occur in neonatal care settings, increasing the risk of hospital-acquired fungal infections, particularly in cases involving multidrug-resistant strains (23). These risks emphasize the importance of early detection and effective management of VVC in pregnant women to reduce complications for both the mother and the neonate.

In Ghana, studies investigating the prevalence and antifungal susceptibility patterns of *Candida* species among pregnant women have been conducted in different regions, revealing notable variations in species distribution and antifungal resistance. Abruquah (24) conducted a study at Komfo Anokye Teaching Hospital (KATH) in Kumasi and reported *C. albicans* as the predominant species, though non-*albicans Candida* species were also present. Antifungal susceptibility testing indicated that amphotericin B was the most effective antifungal agent, with an 87.2% susceptibility rate, whereas some isolates exhibited reduced susceptibility to fluconazole, the most commonly used azole antifungal.

In contrast, a more recent study by Waikhom et al. (15) conducted in Ho found that *N. glabrata*, rather than *C. albicans*, was the predominant causative species. This shift in species distribution is significant, given that NAC species are known to exhibit higher resistance to azole antifungal agents. Antifungal susceptibility testing in the study further highlighted this concern, as resistance to fluconazole was prevalent among *Candida* isolates, whereas voriconazole remained largely effective (15). This differs from the findings in Kumasi by Abruquah (24), where amphotericin B was the most effective antifungal agent, and fluconazole resistance was noted in some but not the majority of isolates.

Additionally, a study by Konadu et al. (25) in Kintampo reported a VVC prevalence of 36.5% among pregnant women but did not conduct species identification or antifungal susceptibility testing, leaving a gap in understanding the regional variations in *Candida* species distribution and resistance profiles. The lack of such data from Kintampo and other regions limits broader comparisons and highlights the need for further studies to establish a comprehensive national profile of *Candida* species and their antifungal susceptibility patterns.

Despite these previous studies, critical gaps remain in the understanding of *Candida* species distribution and antifungal resistance profiles in the Ho municipality. The shift from *C. albicans* dominance in Kumasi (24) to *N. glabrata* dominance in Ho (15) raises concerns about emerging NAC species, particularly given their reduced susceptibility to first-line azole antifungal treatments. Furthermore, variations in antifungal susceptibility patterns across different regions emphasize the need for localized surveillance of resistance trends to guide effective treatment strategies.

This study aimed to determine the prevalence and antifungal susceptibility patterns of *Candida* species among pregnant women attending antenatal clinics in the Ho municipality. The findings provide essential data on species distribution and resistance patterns, thereby informing appropriate therapeutic interventions and contributing to broader efforts to mitigate antifungal resistance in Ghana and beyond.

## MATERIALS AND METHODS

### Study setting and design

A cross-sectional study was conducted from November 2020 to July 2023 at the Ho Teaching Hospital (HTH) and the Ho Municipal Hospital (HMH), which are the major referral health facilities located in Ho, the capital city of the Volta Region.

### Inclusion criteria

Pregnant women at any stage of gestation who visited HTH and HMH during the sampling period for antenatal care and consented to be part of the study were enrolled.

### Exclusion criteria

Pregnant women who were not attending HTH and HMH for antenatal care and/or did not provide informed consent to participate in the study during the sampling period.

### Sample size determination

A total of 500 pregnant women were anticipated to attend the aforementioned health facilities, and this population size was used as the basis for determining the sample size. The sample size calculation was performed using the following formula:


$$n=Z^{2}p\left(1-p\right)/d^{2},$$

where *Z* is the standard score based on the confidence level, *P* is the estimated proportion, and *d* is the margin of error.

Considering an estimated proportion of 21% (24), a confidence interval of 95%, *Z* = 1.96, and 5% margin of error, the sample size calculated was 256. However, due to the challenges of getting all consented participants to partake in the study during the COVID-19 pandemic, data from a total of 205 pregnant women were obtained for inclusion in the study.

### Sample collection and processing

The prospective study was conducted at the antenatal units of Ho Teaching and Ho Municipal Hospitals. Following a detailed explanation of the sampling procedure to the participants, two high vaginal swabs were obtained by a gynecologist or a trained midwife, with sterile cotton-tipped swabs from the consented participants and transported on ice to the Microbiology laboratory of the Ho Teaching Hospital within 1 hour of collection for processing and analysis. One swab was used for culture, and the other was used for a direct smear for Gram staining to detect oval-shaped organisms with buds, pseudohyphae, or hyphae. Colony identification and Gram and urease tests were carried out on the colonies obtained. Yeast-like cells that were urease-negative were subjected to non-PCR methods of speciation, i.e., germ tube test, HiCrome *Candida* differential agar, carbohydrate assimilation or fermentation, and Cornmeal-Tween 80 agar. Using the Kirby-Bauer disk diffusion method, isolates were subjected to fluconazole (10 µg), nystatin (100 IU), itraconazole (10 µg), and clotrimazole (10 µg). The *Candida* isolates were stored in Brain Heart Infusion plus 15% glycerol at −20°C until required for further investigations.

### Culture and fungal identification

The swabs were inoculated on Sabouraud Dextrose Agar (SDA) (HiMedia Laboratories, India) and incubated aerobically at 37°C. The isolated *Candida* colonies on SDA were subcultured onto HiCrome agar (HiMedia Laboratories, India) and incubated at 37°C for 24 hours. The colonies on SDA were also subjected to the germ tube test and sugar assimilation tests for the identification of species. The color of the colonies produced on the HiCrome agar was interpreted, and species were identified per the manufacturer’s chart correlating with sugar assimilation tests.

### Antifungal susceptibility testing

Antifungal susceptibility testing was performed using the disc diffusion method as recommended by the Clinical Laboratory Standards Institute (CLSI) M44A. Standard colony suspensions (0.5 McFarland) (≈1.5 × 10⁸ CFU/mL) were prepared in 5 mL of sterile saline by picking *Candida* colonies from a 24-hour SDA culture plate. The standard colony suspensions were streaked on Muller-Hinton agar (Oxoid, CM0337, UK). The antifungal agents tested were fluconazole disc (10 µg), itraconazole (10 µg), clotrimazole (10 µg), and nystatin (100 IU) antifungal discs (Thermo Scientific Oxoid) as recommended by the CLSI M44A. The plates were incubated aerobically at 37°C and read at 24 hours. The diameters of zones of inhibition were measured for each antifungal disc. Zones of inhibition were interpreted as susceptible (S), susceptible dose-dependent/intermediate (SDD), and resistant (R) according to CLSI standards. Quality control was undertaken by using quality control strains, American Type Culture Collection (ATCC 90028).

### Data management and analysis

Both clinical and laboratory data were extracted and recorded manually into MS Office Excel version 16. Data were exported, coded, and analyzed using Statistical Package for the Social Sciences version 22 (SPSS Inc., Chicago, IL, USA). Categorical data variables were statistically described in frequencies and percentages, while continuous data variables were summarized as mean (standard deviation). The associations between categorical variables were performed using the chi-square test. The *P*-value was considered to be statistically significant if less than 0.05. A univariate logistic regression model was used to identify potential risk factors for VVC. The association between VVC and clinical presentations of study participants was determined using the chi-square test.

## RESULTS

### Socio-demographic data of participants

The socio-demographic data of participants were obtained from the questionnaire administered to them following their consent to participate in the study. The information included their age, education, marital status, and employment status as well as religion and *Candida* infections (Table 1).

**TABLE 1:** General demographic and candidiasis data of the participants (*N* = 205)^*a*^^,^^*b*^

Parameter	Frequency		Percentage (%)
Total respondents	205		100
Age (years)	29.6 ± 5.9	Range (14–43)	
Age group (in years)			
<20	10		4.9
20–29	94		45.9
30–39	95		46.3
40–49	6		2.9
Educational status			
None	5		2.4
Basic	85		41.5
Secondary	60		29.3
Tertiary	55		26.8
Marital status			
Single	22		11.2
Married	183		88.8
Religion			
Muslim	1		0.5
Christian	204		99.5
Employment status			
None	22		10.73
Informal	161		78.54
Formal	22		10.73
*Candida* infection			
Negative	101		49.3
Positive (VVC)	104		50.7
Symptomatic VVC	49		47.1
*C. albicans*	24		49.0
*C. dubliniensis*	1		2.0
*N. glabrata*	6		12.2
*C. tropicalis*	0		0
*C. krusei*	18		36.7
Asymptomatic VVC	55		52.9
*C. albicans*	23		41.8
*C. dubliniensis*	3		5.5
*N. glabrata*	13		23.6
*C. tropicalis*	5		9.1
*C. krusei*	11		20.0

^*a*^ Data are presented as frequency and percentage.

^*b*^ Mean age ± standard deviation.

The study analyzed 205 participants, with an average age of 29.6 ± 5.9 years (14–43). Most participants were in the age range of 20–39, representing 92.2%. Among them, 42% had basic education, 88.8% were married, and 99.5% identified as Christian. Employment was largely informal (78%). Regarding *Candida* infections, 50.7% tested positive for VVC, while 49.3% were negative. Among the symptomatic cases (47.1%), the common species identified were *C. albicans* (49%) and *C. krusei* (36.7%). Among the asymptomatic cases (52.9%), *C. albicans* (41.8%) and *N. glabrata* (23.6%) were the most prevalent.

An association between socio-demographic data and the occurrence of VVC in women showed the following results (Table 2).

**TABLE 2:** Association between socio-demographic characteristics and VVC (*N* = 205)^*a*^

	VVC		OR (unadjusted)	*P*-value for OR
	Negative *n* (%)	Positive *n* (%)		
Total respondents	101 (49.3)	104 (50.7)		
Age group (in years)				
<20	4 (40.0)	6 (60.0)	1	
20–29	47 (50.0)	47 (50.0)	2.508 (0.301–20.887)	0.395
30–39	47 (49.5)	48 (50.5)	1.374 (0.287–6.586)	0.691
40–49	3 (50.0)	3 (50.0)	1.419 (0.299–6.744)	0.660
Educational status				
None	1 (20.0)	4 (80.0)	1	
Basic	44 (51.8)	41 (48.2)	3.722 (0.372–37.184)	0.263
Secondary	30 (50.0)	30 (50.0)	0.780 (0.369–1.650)	0.516
Tertiary	26 (47.3)	29 (52.7)	0.912 (0.420–1.982)	0.816
Marital status				
Single	12 (54.5)	10 (45.5)	1	
Married	89 (48.6)	94 (51.4)	0.722 (0.263–1.979)	0.527
Religion				
Christian	101 (49.5)	103 (50.5)	1	
Muslim	0 (0.0)	1 (100.0)	0	1.000
Employment status				
None	12 (54.5)	10 (45.5)	1	
Formal	10 (45.5)	12 (54.5)	0.746 (0.261–2.133)	0.585
Informal	79 (49.1)	82 (50.9)	1.025 (0.399–2.631)	0.959

^*a*^ Data are presented as frequency and percentage in parentheses. OR, odds ratio.

In Table 2, the association between socio-demographic characteristics and VVC among the 205 respondents indicates no statistically significant relationships. Regarding age, individuals aged 20–29 had an odds ratio (OR) of 2.508 (0.301–20.887) with a *P-*value of 0.395, while those aged 30–39 had an OR of 1.374 (0.287–6.586) with a *P-*value of 0.691, and those aged 40–49 had an OR of 1.419 (0.299-6.744) with a *P-*value of 0.660, all suggesting no significant association. Educational status also showed no meaningful relationship, as individuals with basic education had an OR of 3.722 (0.372–37.184) and a *P-*value of 0.263, while those with secondary and tertiary education had ORs of 0.780 (0.369–1.650) and 0.912 (0.420–1.982) with *P-*values of 0.516 and 0.816, respectively. Marital status showed no influence on VVC, with married individuals having an OR of 0.722 (0.263–1.979) and a *P*-value of 0.527. Similarly, religion was inconclusive, as only one Muslim participant had VVC, resulting in an OR of 0 and a *P*-value of 1.000. Furthermore, employment status showed no significant effect, with formally employed individuals having an OR of 0.746 (0.261–2.133) and a *P*-value of 0.585, while those in informal employment had an OR of 1.025 (0.399–2.631) and a *P*-value of 0.959. Thus, none of the socio-demographic characteristics considered in the study, i.e., age, education, marital status, religion, or employment, was significantly associated with VVC, as all *P*-values exceeded the 0.05 threshold.

Additional information related to the occurrence of VVC, such as participants’ knowledge about VVC, history of miscarriage, previous *Candida* treatment, or whether they had other non-communicable diseases including diabetes, hypertension, vaginal bleeding, or immunocompromised status, was also collected. The *P*-values obtained showed no significant association between the factors aforementioned and VVC (Table 3).

**TABLE 3:** Other information about participants (*N* = 205)^*a*^^,^^*b*^

	Frequency	Percentage	VVC	*P*-value
Heard about *Candida*	167	81.5	84 (50.3)	0.908
Preterm/miscarriage	43	21.0	19 (44.2)	0.391
Antifungal treatment	8	3.9	5 (62.5)	–
Diabetes	3	1.5	3 (100.0)	–
Hypertension	2	1.0	0 (0.0)	–
Bleeding per vagina	2	1.0	2 (100.0)	–
Immunocompromised	2	1.0	0 (0.0)	–

^*a*^ Data of VVC are presented as frequency and percentage in parentheses, with the total frequency exceeding the total number of participants reflecting multiple responses. The *P-*value was obtained using the chi-square test.

^*b*^ "–” indicates that no values were recorded in those spaces.

The *P*-values in Table 3 indicate no statistically significant associations between VVC and the available factors. Specifically, hearing about *Candida* (*P* = 0.908) and experiencing preterm miscarriage (*P* = 0.391) showed no significant correlation with VVC. Other conditions assessed, such as antifungal treatment, diabetes, hypertension, bleeding per vagina, and immunocompromised status, showed no *P*-values, suggesting limited data to determine statistical significance.

### Prevalence of VVC and *Candida* species identified in participants

The prevalence of vulvovaginal *Candida* in pregnant women was ascertained as 104 out of the 205 participants. However, 49.3% showed no fungal growth. Five *Candida* species with varying levels of dominance were identified. Considering the 205 participants in both symptomatic and asymptomatic categories, *C. albicans* was predominant (22.9%) followed by *C. krusei* (14.1%) (Table 4). In addition, Pap smears from the study participants showed the absence of yeast cells in 14.6% of them.

**TABLE 4:** *In vitro* susceptibility patterns of *C. albicans* and non-albicans *Candida* species to fluconazole (10 µg), nystatin (100 IU), itraconazole (10 µg), and clotrimazole (10 µg) (*n* = 104)^*a*^

Antifungal agent	*Candida* species	S *n* (%)	SDD *n* (%)	R *n* (%)	Total (%)
Fluconazole	*C. albicans*	21 (44.7)	1 (2.1)	25 (53.2)	47 (100.0)
	Non*-albicans Candida*	12 (21.1)	11 (19.3)	34 (59.6)	57 (100.0)
	*N. glabrata*	0 (0.0)	11 (57.9)	8 (42.1)	19 (100.0)
	*C. krusei*	9 (31.0)	0 (0.0)	20 (69.0)	29 (100.0)
	*C. tropicalis*	3 (60.0)	0 (0.0)	2 (40.0)	5 (100.0)
	*C. dubliniensis*	0 (0.0)	0 (0.0)	4 (100.0)	4 (100.0)
Nystatin	*C. albicans*	41 (87.2)	0 (0.0)	6 (12.8)	47 (100.0)
	Non*-albicans Candida*	49 (85.9)	1 (1.8)	7 (12.3)	57 (100.0)
	*N. glabrata*	18 (94.7)	0 (0.0)	1 (5.3)	19 (100.0)
	*C. krusei*	23 (79.3)	1 (3.4)	5 (17.2)	29 (100.0)
	*C. tropicalis*	4 (80.0)	0 (0.0)	1 (20.0)	5 (100.0)
	*C. dubliniensis*	4 (100.0)	0 (0.0)	0 (0.0)	4 (100.0)
Itraconazole	*C. albicans*	21 (44.7)	1 (2.1)	25 (53.2)	47 (100.0)
	Non*-albicans Candida*	37 (64.9)	2 (3.5)	18 (31.6)	57 (100.0)
	*N. glabrata*	15 (78.9)	1 (5.3)	3 (15.8)	19 (100.0)
	*C. krusei*	18 (62.1)	1 (3.4)	10 (34.5)	29 (100.0)
	*C. tropicalis*	3 (60.0)	0 (0.0)	2 (40.0)	5 (100.0)
	*C. dubliniensis*	1 (25.0)	0 (0.0)	3 (75.0)	4 (100.0)
Clotrimazole	*C. albicans*	41 (87.2)	5 (10.6)	1 (2.1)	47 (100.0)
	Non-*albicans Candida*	49 (86.0)	6 (10.5)	2 (3.5)	57 (100.0)
	*N. glabrata*	16 (84.2)	3 (15.8)	0 (0.0)	19 (100.0)
	*C. krusei*	25 (86.2)	2 (6.9)	2 (6.9)	29 (100.0)
	*C. tropicalis*	5 (100.0)	0 (0.0)	0 (0.0)	5 (100.0)
	*C. dubliniensis*	3 (75.0)	1 (25.0)	0 (0.0)	4 (100.0)

^*a*^ Data are presented as frequency and percentage in parentheses.

### Pattern of antifungal susceptibility

As summarized in Table 4, the *in vitro* susceptibility patterns of *Candida* species to the four antifungal agents reveal varying degrees of susceptibility and resistance. Clotrimazole and nystatin exhibited the highest susceptibility rates, with 87.2% of *C. albicans* and 86% of non-*albicans Candida* susceptible to clotrimazole, and 87.2% of *C. albicans* and 85.9% of non*-albicans Candida* susceptible to nystatin. Fluconazole showed the highest susceptibility and resistance variation, with only 44.7% of C. *albicans* and 21.1% of non-*albicans Candida* being susceptible. In comparison, 53.2% of *C. albicans* and 59.6% of non-*albicans Candida* were resistant. Among non-*albicans* species, *C. dubliniensis* (100%) and *C. krusei* (69%) showed the highest susceptibility and resistance, respectively, while itraconazole showed moderate effectiveness, with 44.7% of *C. albicans* and 64.9% of non-*albicans Candida* being susceptible.

## DISCUSSION

### Socio-demographic data of participants and VVC

The socio-demographic characteristics of participants in this study provide important insights into the prevalence and risk factors associated with VVC among pregnant women (Table 1). The overall prevalence of VVC among pregnant women was 50.7%, with the highest prevalence observed in the 20–39 age group, accounting for 91.3% of positive cases. Specifically, 22.9% of the total participants in the 20–29 age group and 23.4% in the 30–39 age group tested positive for VVC. However, no statistically significant correlation was found between age and VVC occurrence. Similar patterns have been observed in other studies, such as Tsega and Mekonnen (26), who reported the highest prevalence in women aged 30–36 years (38.5%), and Ali et al. (12), where 71.9% of *Candida* infections were in the 24–30 age group, with a significant age-related association. The findings from Hussen et al. (10) also support this trend, with the highest *Candida* infections occurring in women aged 25–30 years. These similarities reinforce that reproductive-age women are more susceptible to VVC, likely due to hormonal and physiological changes during pregnancy. However, in contrast, a study in southwestern Nigeria found no *Candida* isolates in women above 30 years (27), differing sharply from our findings. This variation may be due to differences in study populations, healthcare access, or environmental factors. Nonetheless, the predominance of VVC among reproductive-age women remains a consistent trend across multiple studies.

The overall prevalence reported in this study is notably higher than that recorded in previous studies conducted in Ghana. Abruquah (24) reported a prevalence of 21% among women attending a gynecological clinic at KATH in Kumasi, while Konadu et al. (25) found a prevalence of 20.2% among pregnant women in the middle belt of Ghana. In a study conducted in the Ho municipality, the same municipality as ours, Waikhom et al. (15) reported a prevalence of 30.7%, with 44.4% of positive cases from the 20–29 age group and 40.7% from the 30–39 age group, closely mirroring the age distribution in our findings but with a lower overall prevalence. The observed increase in prevalence over the years may indicate a rising susceptibility among pregnant women in the region, potentially due to changing lifestyle factors, environmental exposures, or improved detection methods. However, variations in study methodologies, sample sizes, and diagnostic techniques may also account for the observed differences in prevalence.

In terms of education, 42% of the participants had only basic education, while 26.3% had tertiary-level education. There was no statistically significant correlation between education level and the occurrence of VVC in this study. This contrasts with Valiani et al. (28) who found that women with lower educational levels were more susceptible to vaginal infections, suggesting that limited education may lead to inadequate hygienic practices and reduced utilization of health services. Similarly, Disha and Haque (29) emphasized that educational status and awareness about personal hygiene play a significant role in reducing the prevalence of recurrent vaginal infections, such as VVC among women. Thus, women with higher education levels are more likely to adopt better hygiene practices and seek timely medical treatment, which contributes to improved adherence to treatment. In support of this, findings by Abdullah et al. (30) further highlighted the importance of health education interventions, particularly through the use of audio-visual media and educational modules, which play a vital role in improving genital hygiene behaviors and reducing the prevalence of vulvovaginal candidiasis among pregnant women, especially those with limited formal education.

Marital status was another socio-demographic factor assessed in the study. A significant proportion (88.8%) of participants were married, yet marital status was not significantly associated with VVC prevalence. This aligns with Rezk and Alqabbasi (31) who found no significant association between marital status and the type of vaginal infections, including VVC among the participants. However, Waikhom et al. (15) reported that the majority (66.7%) of pregnant women diagnosed with vulvovaginal candidiasis were married. Although the association between marital status and VVC was not statistically significant, the authors acknowledged that married women may have higher exposure to risk factors such as increased sexual activity, which could potentially influence the prevalence of VVC. Nonetheless, marital status remains controversial, as multiple studies have reported varying associations. For instance, an observation by Zeng et al. (32) suggests that marital status alone may not independently predict VVC risk; however, when combined with other factors such as sexual behavior, hygiene practices, sedentary lifestyle, and history of genital infections, it may contribute to stratified risk patterns among women of reproductive age.

Employment status in this study showed that 78% of participants were engaged in informal employment, which could reflect socio-economic disparities affecting healthcare access. Disha and Haque (29) noted that socioeconomic factors, including employment type, may affect access to healthcare services and diagnostic assessment. Thus, pregnant women engaged in informal jobs or from lower socioeconomic backgrounds may have limited time or resources to seek proper medical care, potentially increasing their risk of untreated or recurrent VVC infections during pregnancy. Similarly, Aronsson et al. (33) reported that informal employment correlates with delayed antenatal care visits and lower adherence to healthcare services among pregnant women.

Furthermore, religion was not found to be significantly associated with VVC prevalence. While limited data exist on the influence of religious background on VVC occurrence, current literature suggests that biological and behavioral factors, such as a sedentary lifestyle, consumption of sweet foods and drinks, early sexual initiation, multiple sexual partners, intravaginal douching, and poor vulva cleaning habits, play a more crucial role in determining infection risk (32).

### Prevalence of VVC and *Candida* species identified in participants

The prevalence of VVC among the pregnant women in this study was 50.7%, higher than 30.7% reported by Waikhom et al. (15), in antenatal clinic attendees, while Disha and Haque (29) reported 17%–90% of VVC among pregnant women, further confirming the significant burden of VVC within this population with variations attributed to geographical, socioeconomic, and ethnic factors, and differences in sampling and culturing techniques as well as clinical factors.

*Candida albicans* was the most frequently isolated species in this study, accounting for 22.9% of cases. This finding is consistent with prior studies by Maraki et al. (34), who reported that *C. albicans* remains the predominant *Candida* species responsible for VVC globally. However, this study also acknowledged the presence of non-*albicans Candida* species, including *C. krusei*, *N. glabrata*, *C. tropicalis*, and *C. dubliniensis*. The increasing emergence of NAC species is of concern, as these strains have been reported to exhibit higher antifungal resistance (29). Supporting this observation, Panda et al. (35) noted a significant rise in NAC isolates, particularly *N. glabrata* and *C. krusei*, in pregnant women, emphasizing the challenge they pose in antifungal treatment.

The distribution of *Candida* species in symptomatic and asymptomatic individuals further underscores the complexity of VVC diagnosis. While both symptomatic and asymptomatic cases predominantly involved *C. albicans*, the number of occurrences of NAC species was notably higher among asymptomatic than symptomatic individuals. However, there was an increase in *N. glabrata* and a decrease in *C. krusei* in asymptomatic VVC. Similar observations were made by Tsega and Mekonnen (26), who suggested that asymptomatic colonization with NAC species could serve as a reservoir for persistent infections and potential treatment challenges. This aligns with the findings of Akinosoglou et al. (36), who reported that asymptomatic carriers of NAC species often experience recurrent infections due to delayed or inappropriate treatment.

The detection of asymptomatic VVC cases in this study aligns with findings from Akinosoglou et al. (36), who reported that many pregnant women harbor *Candida* species without showing overt symptoms. This highlights the importance of routine screening, as undiagnosed asymptomatic infections may contribute to vertical transmission during childbirth, increasing the risk of neonatal infections (9, 15). The study by Gedefie et al. (22) further corroborates this, emphasizing that NAC species, including *C. krusei*, constituted 36.7% of maternal vaginal colonization cases, with vertical transmission occurring in 44.9% of neonatal infections. These findings underscore the increasing clinical relevance of NAC species in perinatal fungal infections, particularly in resource-limited environments.

The findings from this study, thus, confirm the high prevalence of VVC among pregnant women, with a notable shift toward NAC species. This trend emphasizes the need for comprehensive diagnostic strategies, including species-level identification and antifungal susceptibility assays, to ensure effective management of VVC in pregnancy. Furthermore, the observed antifungal resistance among NAC species highlights the need for ongoing surveillance and tailored treatment protocols to mitigate the risk of therapeutic failures and associated complications.

### Pattern of antifungal susceptibility

The antifungal susceptibility assay in this study revealed considerable variation in drug efficacy among the different *Candida* species. Clotrimazole and nystatin were the most effective antifungal agents, with high susceptibility rates across all *Candida* species. This finding conforms with Mendling et al. (37), who reported the efficacy of clotrimazole against *Candida* treatment in pregnant women. Similarly, Fan et al. (38) noted that nystatin remained one of the most effective antifungal treatments for VVC with recurrence, particularly in cases involving fluconazole-resistant *Candida* strains. These findings are further supported by Kermani et al. (39), who reported a 10% resistance rate among *C. albicans* isolates, corresponding to a 90% susceptibility to clotrimazole. This reinforces its continued efficacy despite evolving resistance patterns.

Fluconazole and itraconazole exhibited significant resistance, particularly among NAC species. In this study, 69% of *C. krusei* isolates and 100% of *C. dubliniensis* isolates were resistant to fluconazole, consistent with Whaley et al. (40), who reported high resistance rates in NAC species. This pattern of resistance is concerning, as fluconazole remains a commonly used first-line antifungal agent for VVC. Similarly, both Sanguinetti et al. (41) and Borah et al. (42) reported an increasing trend in fluconazole resistance among non-*albicans Candida* species, particularly *C. krusei*. The former attributed this resistance in *N. glabrata* and *C. krusei* to *ERG11* mutations and the overexpression of efflux pumps, while the latter highlighted the role of biofilm formation in fluconazole resistance. These reports suggest that multiple mechanisms contribute to the growing challenge of antifungal resistance in NAC species.

Itraconazole exhibited moderate efficacy, with 44.7% of *C. albicans* and 64.9% of NAC species being susceptible. However, growing antifungal resistance threatens its effectiveness. Bhattacharya et al. (43) identified *ERG11* mutations and efflux pump overexpression as key mechanisms driving azole resistance, including itraconazole. Similarly, Ali et al. (12) reported a 57.6% fluconazole resistance rate among NAC isolates, reinforcing concerns about the diminishing potency of azole-based therapies. While resistance mechanisms between fluconazole and itraconazole may overlap, these findings collectively emphasize the urgent need for enhanced antifungal policy and alternative treatment strategies to combat the rising resistance in *Candida* species.

The emergence of antifungal resistance, particularly among NAC species, demonstrates the need for routine susceptibility assays. Empirical treatment without a susceptibility assay may lead to treatment failure and prolonged infections. Waikhom et al. (15) emphasized the importance of personalized treatment approaches based on susceptibility assays to improve clinical outcomes and mitigate the development of resistance. This concern was raised by Galia et al. (44), who emphasized the need for improved surveillance of antifungal resistance to enhance clinical decision-making and strengthen antifungal management programs, identifying current gaps in monitoring efforts.

### Correlation between VVC and congenital morbidities and mortality among babies

Of the 43 women who experienced preterm births or miscarriages in the study (21% of total respondents), 19 tested positive for VVC (44.2%). These results align with previous studies indicating that VVC may contribute to adverse pregnancy outcomes, including premature rupture of membranes, preterm labor, and neonatal infections (21). This is supported by Rasheed (45), who reported that *Candida* species can adhere to epithelial cells and invade tissues, contributing to infections that may pose risks during pregnancy, including vulvovaginal candidiasis, which requires careful management to prevent potential complications. Moreover, Waikhom et al. (15) emphasized the potential for vertical transmission of *Candida*, particularly affecting preterm neonates with underdeveloped immune systems. Ghaddar et al. (46) similarly reported that untreated maternal VVC could lead to neonatal colonization, which, in severe cases, could progress to invasive fungal infections, underscoring the importance of timely screening and treatment during pregnancy (15, 21).

Recent studies further highlight the emerging impact of antimicrobial resistance in *Candida* species, complicating treatment strategies. A study by Borah et al. (42) observed increased resistance among non*-albicans Candida* species, particularly *N. glabrata* and *C. krusei*, which are less susceptible to conventional azole therapies. This is consistent with the findings of Ali et al. (12), who linked azole-resistant *Candida* strains with persistent infections in pregnant women, thereby increasing the risk of vertical transmission and adverse neonatal outcomes. Similarly, Messina et al. (47) reported that preterm infants born to mothers with *Candida* infections exhibited a higher incidence of respiratory distress syndrome, emphasizing the need for proactive maternal antifungal treatment. Furthermore, Messina et al. (47) found that maternal *Candida* infections correlated with elevated systemic inflammatory markers, which may contribute to fetal distress and growth restrictions. These findings reinforce the need for routine antenatal screening and early intervention strategies to mitigate risks associated with maternal *Candida* infections. Even though this study was limited by its sampling time frame and lacked postnatal follow-ups, its findings align with broader research advocating for stringent maternal-fetal infection control measures. Future longitudinal studies should investigate the long-term impact of maternal VVC on neonatal health outcomes.

### Conclusion

The study highlights that while certain socio-demographic factors, such as age and employment status, may contribute to the prevalence of VVC among pregnant women, they are not definitive predictors. Although most participants were aged 20–39 years, consistent with previous studies indicating higher susceptibility in this age group, variations in education, marital status, and religion did not show significant associations with VVC occurrence. The high prevalence rate of VVC (50.7%) observed in the study underscores the substantial burden of this condition among pregnant women, aligning with global findings. *Candida albicans* remained the predominant species, though the rising prevalence of non-*albicans Candida* species, such as *C. krusei* and *N. glabrata,* is concerning due to their higher resistance to antifungal treatments. Antifungal susceptibility assay conducted in this study revealed that clotrimazole and nystatin were the most effective treatments, while significant resistance was observed against fluconazole and itraconazole, particularly among NAC species. This trend emphasizes the necessity for routine species-level identification and susceptibility assaying to guide effective treatment. Moreover, the study identified an association between maternal VVC and adverse pregnancy outcomes, including preterm births and miscarriages, particularly among women infected with NAC species. These findings reinforce the importance of routine antenatal screening, timely diagnosis, and appropriate antifungal therapy to prevent complications. Generally, while socio-demographic factors may influence healthcare access and treatment adherence, the primary risk factors for VVC and its complications were related to the specific *Candida* species involved and their antifungal resistance profiles. To mitigate these risks, future efforts should focus on and be guided by integrating antenatal care programs, including routine screening, susceptibility assaying, and personalized treatment approaches, alongside broader health education initiatives targeting vulnerable populations. Furthermore, while understanding drug susceptibility and species prevalence is essential in all populations, special considerations must be taken when treating pregnant women due to pharmacological changes during pregnancy and potential safety concerns for the fetus.

### Highlights

- *Candida* species showed significant susceptibility to clotrimazole and nystatin but exhibited resistance to fluconazole and itraconazole.
- Non-*albicans Candida *species could play a role in the development of VVC, potentially complicating treatment protocols.
- Vulvovaginal candidiasis continues to pose a risk during pregnancy, as it can affect women at any stage of gestation.

## Data Availability

Additional data and materials supporting the findings of this study are available from the corresponding author upon request.
